# Prevalence of asthma and its symptoms in Sri Lankan adults

**DOI:** 10.1186/s12889-022-14793-3

**Published:** 2022-12-13

**Authors:** K. D. Gunasekera, W. A. D. L. Amarasiri, U. C. M. Undugodage, H. K. M. S. Silva, A. Sadikeen, W. Gunasinghe, A. Fernando, B. P. R. Perera, A. R. Wickremasinghe

**Affiliations:** 1 Nawaloka Hospitals PLC, Colombo 2, Sri Lanka; 2 Department of Physiology, Faculty of Medicine, University of Colombo, Colombo, Sri Lanka; 3 Department of Physiology, Faculty of Medical Sciences, University of Sri Jayewardenepura, Nugegoda, Sri Lanka; 4 National Hospital of Respiratory Diseases, Welisara, Sri Lanka; 5 National Hospital of Sri Lanka, Colombo 10, Sri Lanka; 6 Colombo South Teaching Hospital, Kalubowila West, Sri Lanka; 7 Department of Rogavijnana, Faculty of Indigenous Medicine, Gampaha Wickramarachchi University of Indigenous Medicine, Yakkala, Sri Lanka; 8 Department of Public Health, Faculty of Medicine, University of Kelaniya, Public Health, Ragama, Sri Lanka

**Keywords:** Adult Asthma, Prevalence, Sri Lanka

## Abstract

**Background:**

Data on adult asthma is scarce in Sri Lanka. The objective of this study was to estimate the prevalence of asthma and its symptoms in adult Sri Lankans.

**Methods:**

A cross-sectional study using a translated version of the European Community Respiratory Health Survey screening questionnaire on subjects $\ge$ 18 years from 7 provinces in Sri Lanka was conducted. The asthma was defined as “wheezing in the past 12 months (current wheeze)”, self-reported asthma attack in the past 12 months or on current asthma medication use.

**Results:**

Among 1872 subjects (45.1% males, 48.8% between 18–44 years of age), the prevalence of current wheeze was 23.9% (95%CI: 22.0%-25.9%), self-reported asthma was 11.8% (95%CI: 10.3%-13.2%) and current asthma medication use was 11.1% (95% CI: 9.6%-12.5%). The prevalences were higher in adults > 44 years, 31.4% positively responded to any of the above questions (95%CI: 29.3%-33.4%) and 60.9% of current wheezers did not report having asthma whilst 38.2% used asthma medication. Among current wheezers, 80.1% had at least one other symptom, cough being the commonest. Those with no current wheeze, self-reported asthma and on current asthma medication use, 30%, 35.9% and 36.6%, respectively, reported at least one other symptom. Smokers comprises 22% current wheezers, 20.6% of self-reported asthmatics and 18.7% of current asthma medication users.

**Conclusions:**

The prevalence of asthma in Sri Lankan adults is higher than the other South Asian countries and higher in the older age group. A significant percentage of symptomatic individuals did not report having asthma or being on medication.

## Introduction

Asthma, a multifactorial chronic inflammatory airway disease, is a serious global health problem affecting all age groups [1]. In 2016, the Global Burden of Disease (GBD) study estimated that approximately 339 million people have asthma, and that the burden of disability is high [2] due to physical, psychological and social effects. It is projected that this number would increase to 400 million by 2025, as countries become more urbanized [3]. Asthma prevalence, severity, and mortality vary by geographic region and economic levels. Asthma prevalence is higher in high income countries, however, most asthma-related mortality occurs in lower middle income countries [4]. Asthma incidence and prevalence differ between children and adults. Asthma often begins in childhood, but some may develop it for the first-time during adulthood. Asthma incidence and prevalence are higher among children. However, asthma-related healthcare use, and mortality are higher in adults [5].

The World Health Survey reported that the global prevalences of doctor diagnosed asthma, clinical/treated asthma and wheezing in adults were 4.3%, 4.5%, and 8.6%, respectively, though this varied by as much as 21-fold amongst 70 countries, with countries like Australia reporting very high prevalences [4]. Much less is known about the prevalence of asthma in middle-aged and older adults. Because the symptoms of asthma are not specific to the disease, they can be confused with those of other respiratory diseases, particularly chronic obstructive pulmonary disease (COPD) in later life [6].

In Sri Lanka, asthma ranked 07th in age-standardized Disability-adjusted life years (DALY) rate per 100,000 in 2019 [7]; diseases of the respiratory system were the third leading cause of hospitalization in Sri Lanka [8].

Though, asthma poses a significant burden on the healthcare system of the country, there is a paucity of data on the prevalence of adult asthma in Sri Lanka. This study was carried out to determine the prevalence of asthma and its symptoms in Sri Lankan adults.

## Methods and materials

This analytical cross-sectional survey was conducted from June to July 2013 in 7 out of 9 provinces of Sri Lanka. The sites comprised urban areas in Colombo, Kandy, Jaffna and Galle districts, rural areas in Kurunegala and Anuradhapura districts and an area in the tea plantations of the Badulla district.

### Sample selection

A cluster sampling method was used. Each cluster was defined as a Medical Officer of Health (MOH) area of a district or a ward in a Municipal Council area. A minimum sample of 196 participants from each cluster was required to estimate a prevalence of current wheezing in 20% of the adult population with a 95% confidence interval ranging from to 17% to 23% and a design effect of 2. In addition to account for differences in population sizes of the districts, additional participants were recruited based on probability proportionate to size. The final sample recruited included 241 from the Kurunegala district, 226 from the Anuradhapura district, 191 from the tea plantations in the Badulla district, 688 from the Colombo district, 137 from the Galle district, 286 from the Jaffna district and 218 from the Kandy district. The Colombo district was oversampled to adjust for a small sample size in the Galle district as both were urban areas.

Participants were selected from the most recent voters list (updated within six months of the survey) maintained by Grama Niladhari officers (government officials responsible for the administrative functions of the smallest administrative unit in the country). As a non-response rate of 20% was expected, additional persons were invited to participate in the study. House visits were made to invite participants and explain the purpose of the study. Invited participants were requested to present themselves at designated points on a given date at a specific time for assessment. The non-response rate was less than expected and all invited participants who presented, were included in the study.

### Data collection tool

The English version of the European Community Respiratory Health Survey (ECHRS) screening questionnaire was translated into Sinhala and Tamil by a group of experts. The translated versions were reviewed by a panel of pulmonologists and language specialists for content and face validity and adjustments were made where necessary. The translated versions were then back translated into English by an independent group of experts and the translated English version was compared with the original ECHRS questionnaire for congruence. The Sinhala and Tamil versions of the questionnaire were pilot tested on 20 subjects from the Colombo district who did not take part in the main study to check familiarity of wording and ease of understanding. One question was slightly rephrased.

### Data collection

Participants who presented themselves on invitation at mobile clinics were explained the objectives of the study and informed written consent was obtained. Interviews were carried out by trained staff using translated, validated versions of the European Community Respiratory Health Survey screening questionnaire [9]. All participants were examined by a physician and given necessary advice if required.

### Definitions

Current wheeze was defined as a positive response to the question “During the last 12 months have you experienced attacks of wheezing or whistling breath?”. Self-reported asthma was defined as a positive response to the question “Have you had an attack of asthma in the last 12 months?”. Current asthma medication use was defined as a positive response to the question “Are you currently taking any medicine (including inhalers, aerosols or tablets) for asthma?”. Current smoking was defined based on a positive response to the question “Do you currently smoke any tobacco products such as cigarettes, cigars, or pipes?”.

An urban area was defined as an area administered by a municipal or urban council. The estate sector was defined as plantations which are 20 acres or more in extent and with ten or more resident labourers. A rural area was defined as an area not categorised as an urban or estate sector area [10]

### Data management and analyses

All completed forms were stored at the Central Chest Clinic, Colombo. Data were entered into a password protected computer. Frequency distributions and descriptive statistics were generated using the Statistical Package for the Social Sciences version 20.0. Comparisons were done using chi-square tests and t-tests.

### Patient and public involvement

Patients were not involved in setting the research question or designing. Study findings are made available to the public through dissemination of the study results in scientific journals and conferences.

### Ethical considerations

Approval was obtained from the Ethics Review Committee of the Faculty of Medicine, University of Kelaniya (Ref No. P5/01/2011). Informed written consent was obtained from all participants. Confidentiality of data was maintained. If participants had a medical problem, they were advised or referred to the nearest chest clinic which provided health care services free of charge.

## Results

A total of 1,872 participants were included in the analysis. The socio-demographic profile of participants is given in Table 1.

**Table 1:** Socio-demographic profile of participants

Gender (n, %)	
Male	845 (45.1%)
Female	1027 (54.9%)
Age (n, %)	
18–44	912 (48.7%)
> 44	960 (51.3%)
Area (n%)	
Urban	644 (34.4%)
Rural^**d**^	1228 (65.6%)
Body mass index category (n, %)^a^,^b^	
Underweight	195 (10.7%)
Normal weight for height	948 (51.8%)
Pre-obese	505 (25.4%)
Obese	181 (9.9%)
Smoking status (n,%)^c^	
Ever smokers	307 (20.4)
Current smokers	245 (13.1)
Never smokers	1198 (79.6)

^a^The current WHO BMI cut-off points of 18·5–24·9 kg/m2 (normal range), > 25 (overweight), 25–29·9 kg/m2 (pre-obese), > 30 kg/m2 (obese) were used

^b^49 missing

^c^367 missing

^d^person from the plantations were included in the rural category

The prevalence of current wheezing defined as ‘wheezing in the last 12 months’ was 23.9% (95% CI: 21.8%-25.7%). Of the current wheezers, 336 (75%, 95% CI: 70%-79%) felt shortness of breath on wheezing and 248 (55.3%, 95% CI: 50.6%-59.9%) admitted that they had wheezing when they didn’t have a cold. Of the current wheezers, 359 (80.1%, CI: 76.4%-83.7%) had at least one other symptom suggestive of asthma, of which cough was the commonest symptom (Table 2). Wheezing only was present in 89 (19.9%, CI:16.2%-23.5%) current wheezers; 175 (39.1%, 95% CI: 34.5%- 43.6%) admitted to having a diagnosis of asthma and 170 (37.9%, 95% CI:33.4%-42.3%) were on current asthma medication.

**Table 2:** Prevalences of asthma symptoms in the past 12 months

	All (*n* = 1872) Number of persons with (and prevalence of: 95% confidence interval (CI) of prevalence of) symptoms in total sample	Number of persons with (and prevalence of: 95% confidence interval (CI) of prevalence of) symptoms among current wheezers (*n* = 448)	Number of persons with (and prevalence of: 95% confidence interval (CI) of prevalence of) symptoms among persons with self-reported physician diagnosed asthma (*n* = 219)	Number of persons with (and prevalence of: 95% confidence interval (CI) of prevalence of) symptoms in persons on current medication for asthma (*n* = 206)
Wheezing	448 (23.9%: 95% CI—21.8%—25.7%)	-	175 (79.9%: 95% CI -74.6% -85.1%)	170 (82.5%: 95% CI—77.3% -87.6%)
Tightness of chest	358 (19.1%: 95% CI—17.3%—20.8%)	180 (40.1%: 95% CI—35.5%—44.6%)	117 (53.4%: 95% CI—46.8%—59.9%)	108 (52.4%: 95% CI -45.5%—59.2%)
Shortness of breath	402 (21.4%: 95% CI—19.5%—23.2%)	207 (46.2%: 95% CI—41.5%—50.8%)	143 (65.2%: 95% CI -58.9%—71.4%)	129 (62.6%: 95% CI -59.2%—69.1%)
Cough	602 (32.1%: 95% CI—29.9%—34.2%)	263 (58.7%: 95% CI—54.1%—63.2%)	148 (67.5%: 95% CI—61.3%—73.6%)	131 (63.5%: 95% CI—56.9%—70.0%)
Nasal symptoms	387 (20.6%: 95% CI—18.7%—22.4%)	172 (38.3%: 95% CI—33.8%—42.7%)	99 (45.2%: CI-38.6–51.7)	96 (46.6%: CI-39.7–53.4)
At least one symptom other than wheezing	900 (48.0%: 95% CI- 45.7%—50.2%)	359 (80.1%: 95% CI—76.4%—83.7%)	192 (87.6%: 95% CI—83.2%—91.9%)	177 (85.9%: 95% CI—81.1%—90.6%)

The prevalence of self-reported physician diagnosed asthma was 11.6% (95% CI: 10.1%-13.0%) and that of current asthma medication use was 11.0% (95% CI: 9.5%-12.4%).

The prevalences of current wheeze, self-reported asthma and that of current asthma medication use in adults aged 18–44 years were 19.6% (95% CI: 17.0%-22.2%), 9.1% (95% CI: 7.3%-11.0%), and 8.2% (95% CI: 6.5%-10.0%), respectively. The prevalence of current wheezing (19.6%; 95%CI: 17.0%-22.2%) was significantly higher than the prevalence of self-reported asthma (9.1%; 95% CI: 7.3%-11.0%) and being on current asthma medication use (8.2%; 95% CI: 6.5%-10.0%); however, the prevalence of self-reported asthma was not different from the prevalence of current asthma medication use. The prevalences of current wheeze, self-reported asthma and that of current asthma medication use in adults aged > 44 years were 28.1% (95% CI: 25.2%-30.9%), 14.2% (95% CI: 12.0%-16.4%), and 13.8% (95% CI: 11.6%-16.0%), respectively.

The prevalences of current wheeze, self-reported asthma and that of current asthma medication were higher in females compared to males. The percentages of females with current wheeze, self-reported asthma and that of current asthma medication were 14.3%, 7.1% and 7.2% respectively. The percentages of males with current wheeze, self-reported asthma and that of current asthma medication were 9.6%, 4.7% and 3.8%, respectively.

The prevalence of other asthma symptoms in the past 12 months in those with current wheezing, self-reported physician diagnosed asthma and on current asthma medication use are given in Table 2.

Of those who did not have wheezing in the past 12 months, 427/1425 (30%) had at least one other symptom suggestive of asthma.

In those who had current wheeze, self-reported asthma and on current asthma medication use, the proportions of current smokers were 22%, 20.6% and 18.7%, respectively; of them 60%, 62.4% and 63.8%, respectively were above 44 years of age. The prevalence of current wheeze was not higher in ever smokers or current smokers, compared to non-smokers.

The prevalence of asthma though higher in rural dwellers was not significantly different from urban dwellers; however, the prevalence of current wheeze was significantly higher in those from rural areas as compared to those from urban areas (Table 3).

**Table 3:** Asthma prevalence by area of residence

	Asthma prevalence (and 95% confidence intervals (CI)) by area of residence	
	Urban (*n* = 1228)	Rural (*n* = 644)^a^
Current wheeze	262 (21.3%; 95%CI:18.7% -23.2%)	186 (28.9%;95% CI:24.5% -31.4%)
Self-reported, physician-diagnosed asthma	140 (11.4%:95% CI:9.2% -12.7%)	81(12.6%;95% CI:9.5%—14.4%)
On current asthma medication use	124 (10.1%;95% CI:8.3% -11.6%)	83 (12.9%; 95% CI:9.5% -14.4%)

^a^Includes the plantation sector workers

## Discussion

This is the first large scale adult asthma prevalence study carried out in seven provinces of Sri Lanka. This cross-sectional questionnaire-based study reports a 23.9% prevalence of current wheezing among adults. The prevalence of current wheeze was lower in those < 44 years of age (19.6%) as compared to adults > 44 years of age (28.1%). Cough was the commonest symptom following wheezing. The prevalence of self-reported physician diagnosed asthma and current asthma medication use was less, reflecting possible self-denial/underreporting of asthma. Persons from rural areas had a significantly higher prevalence of current wheezing as compared to those from urban areas. One fifth of clinically diagnosed asthmatics were smokers.

Even though approximately half of the global asthma population report wheezing in the past 12 months, only a moderate proportion is diagnosed and/or receiving treatment. The highest overall prevalence of asthma has been observed in resource-rich countries but high prevalences are often reported from resource-poor nations as well [1, 4, 11]. In Sri Lanka, the number of deaths related to asthma/ wheezing per 100,000 population in 2019 was 2.6 and the number of hospitalizations were 815.5 per 100,000 population [12]. In 2016, the prevalence of asthma/ wheezing in elderly males was 29.8% and, in females, 29.1% [8]. This study reports a higher prevalence of asthma symptoms than previous reports in Sri Lanka and a higher prevalence among females. In South Asian countries, a review reported that asthma is more prevalent among women in all countries except in India and Nepal [13].

To make comparisons of the prevalence of asthma between different parts of the world, and changes over time, standardized measurements are needed. Use of questionnaires is the most feasible method for large scale surveys. The World Health Survey (WHS study) in 2002–2003, provides the current global status of the burden of asthma and shows that asthma continues to be a major global public health problem. The global survey estimated the prevalence of clinical asthma in adults to be 4.5% worldwide varying by as much as 21-fold across the 70 participating nations. The prevalence of asthma diagnosed by a medical practitioner significantly varied amongst the 70 countries, with the lowest being 0.2% in China and the highest being 21.0% in Australia. Amongst the population living with clinical asthma, almost 25% were current smokers, 50% reported a history of wheezing in the past 12 months, and 20% had never received asthma treatment [4]. Other studies have also reported varying prevalences ranging between 4–20% [14, 15].

Chowgule et al. (1998) report a current wheeze prevalence of 4.1% in adults aged 20–44 years.

Our study was carried out in 2013, utilizing the same three questions used in the WHS survey to diagnose asthma [4]; we report a prevalence of 23%, much higher than the estimated global prevalence of 4.5%, similar to that of Australia and much higher than that reported from other South Asian countries. The prevalences of asthma reported in Bangladesh, India, Nepal, Pakistan, and all South Asian countries, were 5.2%, 6.3%, 4.2%, and 3.7%, respectively [13]. In the Middle East, the observed adjusted prevalence of asthma ranged from 4.4% to 7.6% [16].

Though the WHS survey reported a prevalence of 6.35% and another study using the data of the WHS reported a prevalence of asthma in Sri Lanka as 5.3%, we report a much higher prevalence.

The reasons for the increase in the prevalence of asthma in Sri Lanka in this study could be sub-optimal therapy due to unavailability, lack of awareness, education, effective asthma management programmes and very frequently, denial. Our study revealed that the prevalence of self-reported physician diagnosed asthma and current asthma medication use was less suggesting possible denial of the presence of asthma.

In Sri Lanka, the government provides free healthcare services, including consultation, out- and in-patient care, laboratory investigations and treatment, through a wide network of primary care institutions which can be accessed within 3–5 kms of residential areas. All primary care institutions have qualified physicians who can prescribe medicines for asthma symptoms which are included in the essential medicine list of primary care institutions in Sri Lanka. In addition, internal medicine specialists are located in higher level hospitals and specialists in respiratory medicine are available in specialised chest clinics (*n* = 315) located in different parts of the country [12]. All these institutions provide free healthcare services including medicines. Despite this availability, many persons with asthma are reluctant to access these services for optimal care due to the stigma associated with the diagnosis which, in local terms, may sometimes be construed as disability of a divine nature. A study done in 2007 by the Asian Asthma Patient Coalition, including Sri Lanka, reported that there is stigma attached with asthma and reluctance of asthma patients admitting that they have asthma [17]. It has also been reported that doctors are reluctant to label a child as an “asthmatic” due to the stigma associated with having a chronic condition [18, 19]. Previously it has been reported that denial of asthma in children in Sri Lanka is a possible reason for the differences between current wheezing and prevalence of asthma and on medication for asthma [18].

The fact that the prevalence of asthma and on being on asthma medication being similar is likely to suggest that those reporting asthma are actually physician diagnosed to be on treatment.

When prevalence data from the International Study of Asthma and Allergies in Childhood (ISAAC) [20] were compared with the data from the ECRHS [21], the prevalence estimates in the 13–14 year age group in the ISAAC study were higher. Comparatively, the prevalence of asthma in children and adolescents of Sri Lanka appears to be high as well. However, using the International Study of Asthma and Allergies in Childhood (ISAAC) written questionnaire in Sri Lanka, the country-wide prevalence of current wheeze in 2001 and 2013 among 6–7 year olds was 27.4% and 18.1%, respectively; and among 13–14 year olds, it was 22.5% and 17.8%, respectively [22]. Other studies have reported that the global average of current wheeze among 13–14 year olds was 14.1% and among 6–7 year olds 11.5% [23], lower than the values reported in Sri Lankans.

A limitation of our study is that we did not exclude other possible causes of cough in the study population. However, other studies have reported similar findings [13], with high prevalence of chronic cough.

Population density has increased with higher densities in urban areas. In 2019, the population density of Colombo was 3621persons per square kilometre and is likely to have increased over the years.

The WHS survey revealed that the prevalence of clinical asthma among rural (4.86%) residents is comparable to urban (4.91%) residents in all regions except the Western Pacific [4]. In our study, the prevalence of asthma was higher in rural areas.

Burney et al. suggest that the wide variability of asthma prevalence in populations are unlikely to have important genetic differences and may be suggestive of the preventable nature of asthma. They further describe that this difference is most noticeable in developing countries, where very large increases in asthma prevalence have been observed in urbanized or more westernized areas [9]. In our sample there was no significant difference in the prevalence of self-reported physician diagnosed asthma and on current asthma medication use between urban and rural dwellers. However, among rural dwellers, the prevalence of current wheezing was significantly higher than that in urban residents. It is unlikely that these differences are stemming from disparities in provision of healthcare services as free healthcare is provided through government health centres country wide. In rural areas, the catchment areas of the service centres are large. In this sample, there may have been an overrepresentation of patients from rural areas. Prevalence of current wheezing is also influenced by treatment adherence of the patients which again can be affected by financial affordability, various beliefs and level of education. Moreover, urban areas have more medical officers than rural areas which allows more opportunities for the disease to be detected and treated [24]. Several other factors may contribute to the higher prevalence of wheezing among the rural population. Occupational and environmental exposures associated with rural allergen triggers associated with farming which are abundant in these areas may be a contributing factor. Indoor air pollution due to the use of biomass as fuel for cooking purposes may be another as the practice is common among the rural population [25]. The percentage of households using biomass as a cooking fuel is higher in rural areas (96.3% in estate sector and 84.2% in other rural areas) as compared to households in urban settings (34.6%). It has been reported that late-onset asthma is more prevalent among non-smoking females in rural areas for the same reason [26]. It is also possible that people in rural areas perceive the term “wheezing” differently as they are using the term to describe a variety of breathing symptoms such as shortness of breath due to causes other than asthma. Other than that, factors that indirectly contribute to increased prevalence of the disease such as low income and lack of savings to be utilized for medical needs can play a role in sustenance of the condition thus increasing its prevalence [27].

In our study population, 50% were above 44 years of age. This population could include patients with Chronic Obstructive Pulmonary disease. However, the prevalence in adults less than 45 years (19.6%) was still higher than the value projected for the South Asian region. Abramson et al. (2002) reported that the prevalence of wheezing was 20.5% in a population aged 45–69 years in Victoria, Australia [28]. However, there is a dearth of data on internationally standardized comparisons of asthma prevalence in the elderly. The study conducted by Jarvis et al. (2012) gives asthma prevalences for 19 countries standardized to an European population [29].

We cannot rule out the possibility of inclusion of patients with Asthma–chronic obstructive pulmonary disease overlap syndrome (ACOS) that may be a possible reason for the high prevalence of asthma in young adults. Persistent airflow limitation is a key manifestation in this condition. Moreover, features of both asthma and COPD, even though these conditions are different in terms of patterns of inflammation, the structures affected and the primary anatomical site at which pathological changes occur, are observed in patients with ACOS [30]. Chronic inflammation and airway remodelling are features common to both conditions. These differences between asthma and COPD are clearly observable when young non-smokers with asthma are compared with older smokers with COPD. In our study, the diagnosis of asthma was made based on current wheezing status. Therefore, it is likely that the sample may have included patients with ACOS as well.

22% of the participants with clinical asthma in this sample were smokers which was similar to the global prevalence of smoking (23.5%) [4]. One of the major obstacles in combating the global burden of asthma is the high prevalence of smoking. Approximately, 40% of the participants in South Asian countries have reported regular smoking, especially in Bangladesh and Nepal. The rate of occasional smoking was considerably lower compared to regular smoking except in Sri Lanka where the rate of regular smoking was lowest but the rate of occasional smoking was high. The prevalence of asthma, dyspnoea, and chronic cough was higher among those who engaged in smoking daily or occasionally compared to those who did not [13]. In our study, there were no significant differences in the prevalences of current wheeze, self-reported or physician diagnosed asthma or use of asthma medication between smokers and non-smokers.

Clinical history and demonstration of reversible airway obstruction by spirometry is the gold standard for diagnosing asthma. However, we used a questionnaire-based classification as it was a reasonable, feasible and practical alternative. Utilization of spirometry could provide a more objective assessment of presence of asthma and could further increase its prevalence.

In conclusion, our results indicate that the prevalence of asthma in Sri Lankan adults is high. Further studies need to be undertaken to estimate its burden and identification of preventable risk factors to enable effective management and reduction of further morbidity and mortality.

## Data Availability

The datasets generated and/or analysed during the current study are not publicly available due to the request to do so not being approved by the Ethics Review Committee but are available from the corresponding author on request.

## Abbreviations

DALY: Disability-adjusted life years
GBD: Global Burden of Disease
COPD: Chronic obstructive pulmonary disease
MOH: Medical Officer of Health
ECHRS: European Community Respiratory Health Survey
WHS: World Health Survey
ISAAC: International Study of Asthma and Allergy in Childhood
ACOS: Asthma-COPD Overlap Syndrome
